# Supplementary material for: Elevated interleukin-8 expression by skin fibroblasts as a potential contributor to pain in women with Fabry disease
Source: PLoS One. 2024 Apr 9;19(4):e0300687. doi: 10.1371/journal.pone.0300687 (PMC11003625; doi:10.1371/journal.pone.0300687)
Supplement: S1 Table — (DOCX) [file pone.0300687.s001.docx]

**Supplementary Table 1: Patient population**

| **ID** | **Sex/**  **Age/ Age at diagnosis**  **(yrs)** | **FD-specific treatment** | **Genetic variant** | **GLA activity**  **(normal: 0.4-1.0 nmol/min/mg protein)** | **Age at pain onset** | **Max. NRS** | **IENFD distal calf**  **(fibers/mm)** | **IENFD back th5-level**  **(fibers/mm)** | **QST small fiber impairment** |
| --- | --- | --- | --- | --- | --- | --- | --- | --- | --- |
| **1** | w/45/6 | none | c.1069C>T // p.Q357X | 0.26 | adulthood | “mild“ | 1.4 | 24.3 | normal |
| **2** | w/51/childhood | none | c1069C>T.p.Gln 357X = c.1069C>T // p.Q357* | 0.27 | childhood | 10 | 9.2 | 27.3 | normal |
| **3** | w/26/21 | none | c1069C>T.p.Gln 357X = c.1069C>T // p.Q357* | 0.15 | childhood | 8 | 11 | 36 | elevated cold pain threshold; paradoxical heat senation |
| **4** | w/33/32 | none | c.644A>G // p.N215S | 0.25 | no pain | no pain | 3.5 | 25 | paradoxical heat sensation |
| **5** | w/27/18 | none | c.404C>T // p.A135V | 0.16 | childhood | 9 | 4.1 | 34.3 | normal |
| **6** | w/66/66 | AGAL-A | c.334C>T // p.R112C | 0.28 | adulthood | 5 | 2.1 | 18.4 | elevated cold detection threshold. paradoxial heat sensation |
| **7** | w/46/28 | AGAL-A | c.363del // p.N122Ifs*8 | 0.25 | childhood | 9 | no biopsy | no biopsy | paradoxical heat sensation |
| **8** | w/59/55 | AGAL-A | intron c.1000-10G>A | no data | no pain | no pain | 3.9 | 16.8 | paradoxical heat sensation; elevated cold detection threshold and thermal sensory limen |
| **9** | w/45/40 | Chaperon | c.644A>G // p.N215S | no data | childhood | 8 | 5.9 | 40 | paradoxical heat sensation; elevated cold and warm detection threshold |
| **10** | w/59/53 | AGAL-B | c.354_368del // p.Q119_Y123del | 0.15 | adulthood | 5 | 4.1 | 10.3 | paradoxical heat sensation; elevated cold detection threshold and thermal sensory limen |
| **11** | w/38/37 | Chaperon | c.1184G>C // p.G395A | 0.28 | no  pain | no pain | 5.3 | 12.9 | paradoxical heat sensation |
| **12** | w/61/61 | none | c.350T>G // p.I117S | 0.59 | adulthood | 5 | 2.3 | 15.4 | normal |
| **13** | w/26/9 | none | c.1025G>T // p.R342L | reduced | adulthood | 5 | 7.8 | 26.4 | normal |
| **14** | w/74/64 | none | c427G>A // p.A143T | 0.4 | no  pain | no pain | 7.3 | 1.2 | elevated cold detection threshold; paradoxial heat sensation |
| **15** | m/43/42 | none | c.644A>G // p.N215S | 0.05 | no  pain | no pain | 4.8 | 20 | paradoxical heat sensation |
| **16** | m/62/62 | none | c.644 A>G // p.N215S | 0.06 | no  pain | no pain | 0.5 | 8 | elevated thermal sensory limen |
| **17** | m/30/28 | none | c.784T>C // p.W262R | 0.05 | childhood | 9 | 0.6 | 14 | elevated cold and warm detection threshold; paradoxial heat sensation |
| **18** | m/36/33 | AGAL-B | c. 486G>T // p.W162C | no data | childhood | 8 | 3.2 | 16.7 | normal |
| **19** | m/62/62 | Chaperon | c.644A>G // p.N215S | 0.05 | no pain | no pain | 1.3 | 21.7 | Paradoxical heat sensation; pin prick hyperalgesia |
| **20** | m/19/10 | AGAL-B | c.994dup // p.R332Kfs*7 | 0.02 | childhood | 10 | 5 | 12.8 | elevated cold and warm detection threshold; paradoxial heat sensation |
| **21** | m/21/37 | AGAL-B | c.188G>A // p.C63Y | 0.02 | childhood | 10 | 0 | 9.3 | elevated cold and warm detection threshold |
| **22** | m/55/41 | AGAL-A | c.395 G>A // p.G132E | 0.03 | childhood | 10 | 2.6 | 4.5 | elevated cold and warm detection threshold |
| **23** | m/30/30 | none | c.644A>G // p.N215S | 0.06 | childhood | acral dysesthesias | 5.3 | 14.8 | paradoxical heat sensation; elevated cold and warm detection threshold |
| **24** | m/26/25 | AGAL-A | c.1072_1074del // p.E358del | 0.02 | childhood | 5 | 5.3 | 18.6 | paradoxical heat sensation; elevated warm detection threshold |
| **25** | m/51/37 | AGAL-B | c.1208 del AAG | 0.03 | childhood | 10 | 0 | 19.3 | paradoxical heat sensation; elevated cold and warm detection threshold |
| **26** | m/51/29 | AGAL-B | c. 1021G>A // p.E341K | 0.02 | childhood | 9 | 0.4 | 12.6 | elevated cold and warm detection threshold |
| **27** | m/55/54 | Chaperon | c.644A>G // p.N215S | 0.05 | no pain | no pain | 0.1 | 7.2 | Paradoxial heat sensation |

**Abbreviations:** AGAL-A = agalsidase-alpha; AGAL-B = agalsidase-beta; f = female; FD = Fabry disease; IENFD = intraepidermal nerve fiber density; m = male; Max. = maximum; NRS = numeric rating scale; yrs = years.
