# Supplementary material for: Elevated interleukin-8 expression by skin fibroblasts as a potential contributor to pain in women with Fabry disease
Source: PLoS One. 2024 Apr 9;19(4):e0300687. doi: 10.1371/journal.pone.0300687 (PMC11003625; doi:10.1371/journal.pone.0300687)
Supplement: Attachment — Submitted filename: Hofmann-et-al_PONE_IL8-in-fibroblasts_2023 FINAL.docx [file pone.0300687.s004.docx]

Hofmann, *et. al.* present this manuscript aimed at defining molecular mechanisms in dermal fibroblasts that contribute to pain in Fabry disease. Fabry disease is a lysosomal storage disease arising from X-linked loss-of-function mutations in α-galactosidase A (*GLA*) that frequently results in a small fiber neuropathy and chronic pain the distal extremities. Substantial evidence in the literature demonstrates accumulation of globotriaosylceramide (Gb3), the subtracted for α-galactosidase A, in the dorsal root ganglia contributes to nociception and pain in Fabry disease; however, recent evidence highlights the role for non-neuronal cell types in pain generally (*e.g.*, keratinocytes, fibroblasts, leukocytes, Schwann cells) and in Fabry disease specifically (*e.g.*, Schwann cells, possibly fibroblasts). To determine which cutaneous cell types may contribute to pain in Fabry disease, the authors recruited a cohort male and female patients with Fabry disease and a cohort of controls from their unaffected family members and friends, cultured and characterized keratinocytes and dermal fibroblasts from these cohorts, and assessed the effects of conditioned media from dermal fibroblasts or interleukin-8 (IL-8) on intrinsic excitability of induced pluripotent stem cells (iPSCs) differentiated into sensory neuron-like cells. The authors observe increased Gb3 accumulation in fibroblasts, but not keratinocytes, from Fabry disease patients, and, thus, focus the reset of their analyses on dermal fibroblasts. The authors also find decreased expression of TRPV1 and NaV1.7 and increased expression of KCNN4 and Il-8 in dermal fibroblasts derived from Fabry disease. The authors also report reduced rheobase (current threshold to evoke a single action potential) in iPSC-derived sensory-like neurons derived from control and a male patient with Fabry disease exposed to conditioned media from dermal fibroblasts derived from a female Fabry disease patient or IL8. From this, the authors conclude that Gb3 accumulation in dermal fibroblasts alters ion channel and cytokine expression, thereby sensitizing nociceptors and contributing to pain in Fabry disease in a sex-specific manner.

This study is quite important and valuable because it is extremely difficult to get tissue samples from Fabry patients and here the authors have conducted a body of work on these patient tissues. Very few, if any other labs, could obtain these tissues and perform these studies; these studies are critical for translating findings in rodent models of Fabry disease. The idea that dermal fibroblasts could contribute to nociception and pain in Fabry disease is meritorious and warrants further study. Overall, I like this study a lot and think this is an appropriate venue for this study.

That said, there are several issues with the data and interpretation that I think should be addressed before publication to make a very robust, impactful publication.

Major revisions:

1) Not all data reported are presented. Specifically:
   a) Patient demographics are not presented in the manuscript.
   b) IENF density measurements are referred to in line 233-234, yet not presented anywhere in the manuscript. Given how these data are described in this section, there may be issues with IENFD analysis as well (see below).
   c) In line 249, the authors write that “NaV1.8 and TRPM8 were not expressed (data not shown).” The authors should present this in their figures.
   d) **Figure 4** presents electrophysiological analysis of iPSC-derived sensory-like neurons dosed with either IL-8 or dermal fibroblast conditioned media from controls, male Fabry disease patients, or female Fabry disease patients. However, the current figure only presents panels for action potential amplitude or rheobase from iPSC-derived sensory-like neurons incubated with conditioned media from control or female patient-derived dermal fibroblasts. Moreover, the authors refer to **Figure 4C** in line 280, yet there is no **C** panel presented in **Figure 4**.
2) The results as presented lack appropriate controls for sex as a biological variable. The authors are right in looking at sex as a potential variable in patients with Fabry disease, but there is no account for potential effects of sex in control fibroblasts. There is substantial rationale that sex in unaffected, healthy patients affects gene expression and release of factors into media. Because sex is not treated as an independent variable, the authors should not claim that the results here are an interaction of sex and disease state (*e.g.*, a sexually dimorphic factor of Fabry disease) or simply additive main effects of sex and disease.
   a) The point above also applies to the sex of iPSCs used in these studies. The authors provide strong rationale for only using iPSC-derived sensory-like neurons from a male Fabry disease patient due to availability, yet in the same preprint where they first describe the lines used herein, they developed a female Fabry patient iPSC-derived sensory neuron-like line (FD-3). The use of only male Fabry disease iPSC lines requires stronger justification. Further, the sex of the control iPSC line is not clearly indicated.
3) There is insufficient detail in the Experimental Methods section:
   a) In describing quantification of IENF density, the authors are right in citing Lauria, *et. al.* (2010); however, they should briefly describe how IENFs were quantified herein instead of writing “Intraepidermal nerve fibers were quantified according to published rules,” as various methods of IENF quantification have been published, and these variations in quantification can lead to different results.
      i) The authors must clarify how these data were analyzed. In their results (lines 223-234), the authors write that only a proportion of patients demonstrated reduced IENFD. If this was determined relative to the control cohort presented in this paper (this appears likely given the methods section detailing use of skin punch biopsies) this analysis is not standard and may be inappropriate. Instead, the authors should compare IENFD between patient and control cohorts by the appropriate parametric or nonparametric test (include representative images and graphical presentation). If the authors instead compared IENFD of patients to age- and sex-adjusted normative values, the authors should explicitly state and cite the source of those values and include a description of how experimental values were determined to be reduced relative to those normative values.
   b) It is unclear how many passages of human-derived keratinocytes and dermal fibroblasts were used in these studies. This should be included in the cell culture section of the methods.
   c) The gene expression analysis section of the methods requires the additional detail. As presented, replication of these methods would not be possible.
      i) It is unclear from the methods whether extracted RNA was quantified or assessed for purity and integrity by UV spectrophotometry (*e.g.*, by a NanoDrop, which is standard in the field).
      ii) The authors do not indicate that they treated their RNA samples with DNase, which is essential to avoid amplifying genomic DNA in their qPCR.
      iii) It is not clear how much mRNA was used in the reverse transcriptase reaction, nor do the methods state how much cDNA was loaded for the qPCR reaction.
      iv) Primers or probe sequences available in a supplementary table.
   d) The authors description of the generation and characterization of iPSC-derived sensory-like neurons in this manuscript is insufficient and the following should be addressed:
      i) The description of the generating these cell lines is cited, but the specific citation for generating control, FD-1 and FD-2 cell lines is a preprint that has yet to undergo peer review. As such, the use of these cells here warrants a more in-depth description of the generation and characterization of these cells. Additionally, FD-2 (a Fabry disease sensory neuron-like line derived from a patient without pain) is not used in this manuscript. In the initial preprint cited here, the FD-2 and control lines were generated with the reprogramming cocktail over the same time course, while FD-1 used a shorter timecourse.
      ii) The authors initially refer to these cell lines as “nociceptive neurons” (line 182-184). It isn’t clear that these are nociceptors. Through the rest of the manuscript, the authors refer to these lines as “sensory neurons”, which is more accurate.
   e) In the patch-clamp analysis section of their methods, the authors do not indicate how many cells were recorded for each media condition.
4) The authors write in the “Statistical analysis” section of the Methods that the Kruskal-Wallis test was used for non-parametric data (followed by Dunn’s multiple comparisons as a *post hoc*, both gene expression and electrophysiological analyses). As no statistical tests are presented for parametric data in this section, it is assumed that Kruskal-Wallis is the main test used herein. The Kruskal-Wallis test is appropriate for nonparametric analyses with a single independent variable (akin to a one-way ANOVA for parametric data). Notwithstanding including sex as an independent variable throughout the manuscript (see **Major Revision 2**), **Figure 4A-B** present electrophysiological data with at least two independent variables (*e.g.*, control vs Fabry disease iPSC-derived sensory-like neurons and media treatments), rendering the Kruskal-Wallis test inappropriate. In all cases where the data are nonparametric and include more than a single independent variable, the authors should use the Friedmann’s test. If the data are parametric, the authors should use a 2- or 3-way ANOVA as appropriate.
5) The authors conclude that the mechanisms they uncover are Gb3-dependent, but this is not necessarily true. Recent evidence suggests Gb3-independent roles of *Gla*-deficiency in a zebrafish model of Fabry disease (Elsaid, *et. al.*, 2023, *J Trans Med*). The authors must at least discuss the possibility of Gb3-independent mechanisms driving the changes observed herein.
   a) Additionally, in this analysis, the authors should consider that keratinocytes, even though they may not accumulate Gb3 in culture, may still play a role in increased pain and nociception in Fabry disease through Gb3-independent consequences of *GLA*-deficiency.
6) The authors could provide a stronger explanation for why changes in ion channel expression—and especially decreased expression of sodium and calcium channels with concomitant increase in potassium channels—in fibroblasts could be a mechanism driving nociceptor sensitization or pain. The specific changes seen herein suggest that fibroblasts might be less excitable. Why would this lead to increased neuronal excitability? As it stands, the expression data for these channels is purely correlative.
7) In the title and throughout the manuscript, the authors describe their proposed mechanism as a driver of pain in severely affected women with Fabry disease. But the authors do not present any data on pain in their cohorts nor criterion for considering a patient’s pain severe. This needs to be remedied—either by including such data and criterion, or by softening the language.

Minor revisions:

1) For PCR analyses, the authors should use the appropriate gene names (*e.g.,* NaV1.7 is *SCN9A*, you are not looking at the protein product of that gene, but it’s transcript). As a more central example, the gene abbreviation of α-galactosidase A for humans is not *α-GAL*, but *GLA*.
   a) Conversely, at some point in the discussion, the authors should mention that *KCNN4* encodes the SK/IK channel. Please do not remove all mention of the functional protein product of these genes (NaV1.7); rather, when talking about gene expression, use the gene name. When talking about the channel, refer to the protein name. This will improve readability and accessibility.
2) While the authors never directly compare the accumulation of Gb3 in dermal fibroblasts to keratinocytes, they should highlight that this comparison is not possible from their current data. There are two reasons for this:
   a) Accumulation of Gb3 was not quantified between these cell types.
   b) The STX used to stain for Gb3 in keratinocytes and fibroblasts cannot be directly compared, as they do not use the same fluorophore.
3) The authors should refine their language in the second paragraph of their patch-clamp analysis section (lines 203-211) to more clearly indicate that all patient conditioned media samples were used as individual replicates.
4) The authors should better cite key contributors to the notion that several types of cutaneous cells are known contributors to nociception and somatosensation. Only one of the cited works on fibroblasts (Kreß, *et. al.* (2022), *PAIN*) directly shows any role for fibroblasts in neuronal excitability. The role for fibroblast control of nociception or pain has not been cited.
5) The Summary **Figure 5** is very crudely drawn and should be made more professional. Additionally, it is not immediately clear what the figure is trying to convey, and clarity could be improved with arrows indicating the flow of ideas.
6) The senior author has previously published that fibroblast-derived conditioned media from patients with small fiber neuropathy sensitizes nociceptors. The authors should better place the results of their current manuscript in the context of their previous findings. This will strengthen the paper.
7) In **Figure 4B**, the data indicate that even control-derived dermal fibroblast conditioned media decreases the rheobase of Fabry patient iPSC-derived sensory-like neurons. In fact, this result is not significantly different from the decrease in rheobase evoked from female patient-derived dermal fibroblast conditioned media. The authors should comment on this in their discussion.
8) Was conditioned media collected in serum-containing or serum-free media? This can have substantial effects on the stability of factors in the media, and the results of placing these conditioned media on other cell types.
9) The authors should read through the current manuscript again for grammatical errors, run-on sentences, and clarity.
